# The association between survey timing and patient-reported experiences with hospitals: results of a national postal survey

**DOI:** 10.1186/1471-2288-12-13

**Published:** 2012-02-15

**Authors:** Oyvind A Bjertnaes

**Affiliations:** 1 Department for Quality Measurement and Patient Safety, Norwegian Knowledge Centre for the Health Services, Oslo, Norway

## Abstract

**Background:**

Research on the effect of survey timing on patient-reported experiences and patient satisfaction with health services has produced contradictory results. The objective of this study was thus to assess the association between survey timing and patient-reported experiences with hospitals.

**Methods:**

Secondary analyses of a national inpatient experience survey including 63 hospitals in the 5 health regions in Norway during the autumn of 2006. 10,912 (45%) patients answered a postal questionnaire after their discharge from hospital. Non-respondents were sent a reminder after 4 weeks. Multilevel linear regression analysis was used to assess the association between survey timing and patient-reported experiences, both bivariate analysis and multivariate analysis controlling for other predictors of patient experiences.

**Results:**

Multivariate multilevel regression analysis revealed that survey time was significantly and negatively related to three of six patient-reported experience scales: doctor services (Beta = -0.424, *p*< 0.05), information about examinations (Beta = -0.566, *p *< 0.05) and organization (Beta = -0.528, *p *< 0.05). Patient age, self-perceived health and type of admission were significantly related to all patient-reported experience scales (better experiences with higher age, better health and routine admission), and all other predictors had at least one significant association with patient-reported experiences.

**Conclusions:**

Survey time was significantly and negatively related to three of the six scales for patient-reported experiences with hospitals. Large differences in survey time across hospitals could be problematic for between-hospital comparisons, implying that survey time should be considered as a potential adjustment factor. More research is needed on this topic, including studies with other population groups, other data collection modes and a longer time span.

## Background

Patient experiences are an important part of health-care quality [1,2]. Surveys are frequently used to measure patient experiences and satisfaction with health care [3,4], but their value is subject to several methodological challenges. One particular methodological challenge relates to the time it takes from the health-care encounter to the patient receives the questionnaire. Questionnaires might be distributed immediately after a healthcare encounter, a short time after the encounter or a long time after the encounter. Since factors like clinical and health-related quality-of-life outcomes might produce different patient evaluations depending on when the survey is applied to patients [3], decisions about survey time might have substantial effects. Following a longitudinal study, two different theoretical models for patient satisfaction were suggested: (i) an immediate post-visit satisfaction model that includes demographics, patient expectations, patient functioning and patient-doctor interaction; and (ii) a model for 2-week/3-month satisfaction that includes demographics, expectations, patient functioning and symptom improvement [5]. This suggests that the results of evaluations of patients would vary with the survey time point.

Several studies have investigated the association between survey timing and patient evaluation [6-16], and most of them found that patient evaluation is poorer when measured at a longer time after the encounter [6-9,11,14-16]. However, a closer investigation of these studies shows that in all except one [11], the data collection mode changed between the different measurements; the aforementioned timing effects might therefore have been due to changes in data collection mode. In fact, the best-designed study concerning the association between survey timing and patient satisfaction found little association between survey timing and patient satisfaction measures [12]. That study grouped patients into three different mailing intervals: 1, 5 and 9 weeks after discharge. All groups received and completed a postal survey at home, and so the data-collection mode was standardized between the groups.

The objective of this study was to assess the association between survey timing and patient-reported experiences with hospitals. The Norwegian Knowledge Centre for the Health Services conducted a national postal patient experience survey among adult inpatients discharged from Norwegian hospitals in 2006. The data set included survey data, administrative data from the hospitals including discharge dates, and practical survey variables including the dates for first postal mailing and for response registration in the Knowledge Centre. The availability of these data made it possible to assess the association between survey timing and patient-reported experiences while simultaneously controlling for other known predictors of patient experiences.

## Methods

### Data

The national survey included adult inpatients discharged from Norwegian hospitals between September 1 and November 23, 2006. The response rate to the survey was 45%, with responses being received from 10,912 patients. In total, 24,141 patients were included in the study; 345 patients were not eligible. The study is described in more detail in another publication [17].

The Norwegian Regional Committee for Medical Research Ethics, the Data Inspectorate and the Norwegian Directorate of Health and Social Affairs approved the survey.

### Questionnaire

The questionnaire comprised 29 items about patient experiences and satisfaction, 14 questions about quality of life and 10 background questions. The patient experience questions were based on the Patient Experiences Questionnaire [18], but the response scale was changed to improve data quality [19]. For 27 of the 29 experience items, a five-point response format was used, ranging from "not at all" to "to a very large extent" The national report used the following six scales with good evidence for data quality, reliability and validity [20]: doctor services (three items), nursing services (four items), information about examinations (two items), organization (three items), hospital and equipment (two items) and contact with next of kin (two items). These scales were used in the present study. Scale scores were transformed to a scale of 0-100, where 100 is the best possible rating.

### Statistical analysis

Patients discharged from one hospital department to another were included in the national survey. For these patients we only saved the latest discharge date, and consequently it is inappropriate to use the discharge date in the computation of survey time. Therefore, patients with more than one department stay were excluded from this study (*n *= 362).

The survey-time variable was computed as the difference between the date of the first postal mailing and the discharge date. The survey time was 11.8 ± 5.7 days (mean ± SD; minimum: 1 day; maximum: 41 days). According to the protocol, the survey time should have been 1-15 days, but delays in transfers from hospitals resulted in a greater variation. Survey time is a continuous variable and was included as such in the regression analysis described below. In bivariate analysis, the survey-time variable was grouped by week, which gave large groups for statistical comparisons. These bivariate analyses are secondary and no other tests were conducted to assess the appropriateness of this grouping. The survey-time variable was divided into the following groups: ≤ 1 week, 1-2 weeks, 2-3 weeks and > 3 weeks. Survey-time groups were compared across six variables: gender, age, education, self-perceived health, admission type and number of admissions in the previous 2 years. Pearson's chi-square was used for statistical testing, except for age, for which one-way analysis of variance (ANOVA) was used.

Response time has been shown to be associated with patient-reported experiences [21]. Response time was computed as the difference between the dates of response registration and first postal mailing. This variable is influenced by how rapidly each individual responded to the questionnaire, and was included as a covariate in the regression models described below. The main focus of the regression analysis was the association between survey timing and the level of patient-reported experiences, adjusted for all the other predictors including response time.

Multilevel linear regression analysis was used to assess the association between survey timing and the six patient experience scales, both bivariate analysis and multivariate analysis controlling for gender, age, self-perceived health, education, admission type, number of admissions in the previous 2 years, response time and hospital. Patient clustering within hospitals might inflate *t *values in ordinary linear regression models so as to produce a type I error, which was the reason for using multilevel regression. The multilevel model divides the total variance in patient-reported experiences into variance at the hospital (macro) level versus the patient (micro) level. The hospitals were included as random intercepts, and all variables from the ordinary regression as fixed effects at the patient level. Standardized variables at level 1 were used in the regression; consequently, standardized regression coefficients were computed. SPSS version 15.0 was used for statistical analyses.

## Results

Three of the six background variables differed significantly across the survey-time groups (Table 1): gender (*p *< 0.05), self-perceived health (*p *< 0.05) and number of admissions in the previous 2 years (*p *< 0.05). Bivariate multilevel regression analysis showed that survey time was significantly and negatively related to three of six patient-reported experience scales: organization (Beta = 0.528 *p *< 0.05), information about examinations (Beta = -0.631, *p *< 0.01) and contact with next of kin (Beta = -0.669, *p *< 0.05) (Table 2).

**Table 1:** Background variables for the survey-time groups (*n *= 10,717)

	Percentage of al respondents	Time between discharge and posting the questionnaire				
		**≤ 1 week** **(*n *= 1,977)**	**1-2 weeks** **(*n *= 4,810)**		**2-3 weeks** **(*n *= 3,290)**	**> 3 weeks** **(*n *= 640)**

*Gender *(%)						
Male	46.7	44.6	46.7		47.1	51.6
Female	53.3	55.4	53.3		52.9	48.4
*p*^a^				*		
Age, mean (years)	60.9	60.9	60.9		60.9	60.3
*p^a^*				ns		
*Education *(%)						
Primary school and lower secondary school	34.0	34.1	34.4		33.5	33.2
Upper secondary school	38.9	38.8	38.2		39.8	40.7
University/college for < 4 years	18.3	19.1	18.2		18.2	17.8
University/college for ≥ 4 years	8.7	8.0	9.2		8.6	8.3
*p*^a^				ns		
*Self-perceived health *(%)						
Excellent	5.3	4.9	5.2		5.7	5.2
Very good	16.2	15.6	16.8		16.1	13.8
Good	33.5	33.0	34.5		33.3	28.6
Fairly good	31.3	31.8	30.1		31.8	35.6
Poor	13.8	14.8	13.4		13.1	16.7
*p*^a^				*		
*Admission type *(%)						
Emergency	50.5	50.5	51.0		49.5	51.6
Routine	49.5	49.5	49.0		50.5	48.4
*p*^a^				ns		
*Number of admissions in the previous 2 years *(%)						
1	46.9	44.7	48.5		46.5	42.6
2	25.4	26.2	24.8		25.7	26.8
3-5	21.2	22.3	21.0		20.4	24.2
6-10	4.5	4.6	4.1		5.0	4.4
> 10	2.0	2.2	1.6		2.4	2.0
*p*^a^				*		

^a^Chi-square tests, except for age (one-way ANOVA). *p < 0.05; ns, not significant

**Table 2:** Bivariate multilevel regression models: associations between survey-time and patient-reported experience scales

	Beta	*p*
Doctor services	-0.355	ns
Nursing services	-0.301	ns
Information about examinations	-0.631	**
Organization	-0.528	*
Hospital and equipment	-0.234	ns
Contact with next of kin	-0.669	*

**p < 0.01; *p < 0.05; ns, not significant

Multilevel regression analysis revealed that several background variables were significantly associated with patient-reported experiences (Table 3). Survey time was significantly related to three of the six patient-reported experience scales: doctor services (Beta = -0.424, *p *< 0.05), information about examinations (Beta = -0.566, *p *< 0.05) and organization (Beta = -0.528, *p *< 0.05). All associations were negative, indicating that the patient-reported experience scores declined with increasing survey time. Patient age, self-perceived health and type of admission were significantly related to all patient-reported experience scales (better experiences with higher age, better health and routine admission), and all other predictors had at least one significant association with patient-reported experiences.

**Table 3:** Multilevel linear regression models: associations between independent variables and patient-reported experience scales

	Doctor services		Nursing services		Information about examinations		Organization		Hospital and equipment		Contact with next of kin	
	**Beta**	***p ***	**Beta**	***p ***	**Beta**	***p ***	**Beta**	***p ***	**Beta**	***p ***	**Beta**	***p ***
Survey time	-0.424	*	-0.330	ns	-0.566	*	-0.528	*	-0.276	ns	-0.497	ns
Response time	-1.21	***	-1.21	***	-1.04	***	-0.858	***	-0.644	**	-0.432	ns
Male (vs. female)	0.090	ns	0.808	***	0.038	ns	0.406	ns	0.411	*	0.496	ns
Age	1.89	***	1.25	***	1.79	***	1.67	***	1.03	***	2.41	***
Self-perceived health	-4.01	***	-3.44	***	-4.70	***	-3.63	***	-2.66	***	-3.64	***
*Education *(vs. primary school):												
Upper secondary school	0.221	ns	-0.152	ns	-0.334	ns	-0.588	*	-0.533	*	-0.252	ns
University/college for < 4 years	-0.239	ns	-0.691	***	-0.589	*	-1.72	***	-1.46	***	-0.592	ns
University/college for ≥ 4 years	-0.045	ns	-0.613	**	-0.405	ns	-1.80	***	-0.772	***	-0.697	*
Routine admission (vs. emergency)	1.48	***	0.844	***	2.36	***	2.49	***	0.455	*	0.875	**
Number of admissions in the previous 2 years	-0.775	***	-0.793	***	-0.230	ns	-0.473	*	-0.656	**	-0.543	*

***p < 0.001; **p < 0.01; *p < 0.05; ns, not significant

## Discussion

Research on the effect of survey timing on patients' evaluations of health services has produced contradictory results [3]. This study found that patients report worse experiences for 3 of 6 patient-reported experience scales when survey time is longer. Individual response time was also negatively related to patient-reported experiences, so regardless of reason increasing time since discharge seems to result in poorer patient-experience scores.

Studies assessing the effect of survey time need to standardize the data-collection mode in order to avoid confounding time and mode effects. Almost all studies showing a worsening in patient evaluation over time changed the data-collection mode between the different measurements [6-9,11,14-16]. Therefore, the timing effects might be related to the mode change rather than being actual timing effects. The current study standardized the data-collection mode and found a significant association between survey time and patient-reported experiences for three of the six scales. This is in line with the aforementioned studies, but contradicts another study from Switzerland in which the data-collection mode was also standardized [12]. However, the Swiss study only included one hospital, a specific patient group and a relatively small sample. Consideration of all of the available data suggests that there is a negative association between survey timing and patient-reported experiences and satisfaction. However, more research is needed including studies with other population groups, other data-collection modes and a longer time span.

The first limitation of the current study relates to the distribution of the survey-time variable. The data-collection protocol means that most patients were sent a questionnaire 0-3 weeks after discharge, with a maximum of 41 days for individual patients. It would be useful to have knowledge about the potential effects of a longer time span, such as the effects of a model with surveys sent 1 month versus 2 months after discharge. The second potential limitation of the current study is the response rate. In general, postal surveys have lower response rates than other data collection modes [3]. The response rate in the current study is in line with other Norwegian national patient experience surveys. Non-response bias occurs when the main variables differ systematically between respondents and respondents [22]. In our study, the main question relates to differences between respondent groups, and hence non-response bias is of less concern. However, response rates might be lower in surveys with a longer time between discharge and postal mailing [12,13]. Consequently, the association between survey timing and the response rate is an important consideration when designing data-collection procedures. A Swiss study found that the response rate was significantly lower for the 9-week group [12] but not for the 5-week group, indicating that satisfactory results regarding response rate can be achieved with surveys mailed 0-5 weeks after discharge.

A third possible limitation of the study concerns its observational research design, causing uncertainty related to potential confounding variables. The gold standard for effect research is randomized trials, in which the aim is for only random variations to exist between study groups and for there to be a direct link between intervention and effect. However, a multicentre randomized trial on this topic would present large practical and methodological challenges, both regarding which time frames to use (intervention) and how to apply sample frames and randomization across hospitals. Another suitable design could have been a longitudinal approach, but that was not possible in this study. The present study adjusted for the most important sociodemographic predictors of patient experiences and patient satisfaction, reducing the probability of confounding effects related to variables not included. The study was based on data from all hospitals in Norway, and the survey-time variable was registered and analyzed as a continuous variable at the individual level. The former feature increased the external relevance of the study, and the latter gave the opportunity to use survey time in days in the analysis, providing more detailed information than only groups based on, say, weeks or months.

## Conclusions

Survey time was significantly and negatively related to three of the six scales for patient-reported experiences with hospitals. Large-scale hospital comparisons of patient-reported experiences should consider survey time as an adjustment factor if it is not standardized across hospitals. The generalizability of the survey-time effect to other topics and other modes is uncertain, but a negative association has been found in most of the other studies referenced, including patient populations in primary care and hospital in- and outpatient care. However, more high-quality research on this topic is needed, including studies with other population groups, other data collection modes and a longer time span.

## Competing interests

The author declares that they have no competing interests.

## Authors' contributions

O.A.B. planned the paper, carried out the statistical analysis, and drafted and finalized the paper.

## Pre-publication history

The pre-publication history for this paper can be accessed here:

http://www.biomedcentral.com/1471-2288/12/13/prepub

## References

[B1] Donabedian A The quality of care: how can it be assessed? JAMA 1988 260 1743 1748 10.1001/jama.1988.03410120089033 3045356

[B2] Kelly E Hurst J Health care quality indicators project: conceptual framework paper OECD Health Working Papers 23-2006 OECD, Paris

[B3] Crow R Gage H Hampson S Hart J Kimber A Storey T Thomas H The measurement of satisfaction with healthcare: implications for practice from a systematic review of the literature Health Technol Assess 2002 6 1 244 12925269 10.3310/hta6320

[B4] Garratt AM Solheim E Danielsen K National and cross-national surveys of patient experiences: a structured review Norwegian Knowledge Centre for the Health Services, Oslo Report 7-2008 29320000

[B5] Jackson JL Chamberlin J Kroenke K Predictors of patient satisfaction Soc Sci Med 2001 52 609 620 10.1016/S0277-9536(00)00164-7 11206657

[B6] Bowman MA Herndon A Sharp PC Dignan MB Assessment of the patient-doctor interaction scale for measuring patient satisfaction Patient Educ Couns 1992 19 75 80 10.1016/0738-3991(92)90103-P 1298951

[B7] Savage R Armstrong D Effects of a general practitioner's consulting style on patients' satisfaction: a controlled study BMJ 1990 301 968 970 10.1136/bmj.301.6758.968 2282112 PMC1664199

[B8] Stevens M Reininga IH Boss NA van Horn JR Patient satisfaction at and after discharge: effect of a time lag Patient Educ Couns 2006 60 241 245 10.1016/j.pec.2005.01.011 16253466

[B9] Lemos P Pinto A Morais G Pereira J Loureiro R Teixeira S Nunes CS Patient satisfaction following day surgery J Clin Anesth 2009 21 200 205 10.1016/j.jclinane.2008.08.016 19464614

[B10] Taylor NG Tollafield DR Rees S Does patient satisfaction with foot surgery change over time? Foot (Edinb) 2008 18 68 74 10.1016/j.foot.2008.01.003 20307415

[B11] Bendall-Lyon D Powers TL Swan JE Time does not heal all wounds: patients report lower satisfaction levels as time goes by Mark Health Serv 2001 21 10 14 11525135

[B12] Saal D Nuebling M Husemann Y Heidegger T Effect of timing on the response to postal questionnaires concerning satisfaction with anaesthesia care Br J Anaesth 2005 94 206 210 10.1093/bja/aei024 15542538

[B13] Brédart A Razavi D Robertson C Brignone S Fonzo D Petit JY de Haes JC Timing of patient satisfaction assessment: effect on questionnaire acceptability, completeness of data, reliability and variability of scores Patient Educ Couns 2002 46 131 136 10.1016/S0738-3991(01)00152-5 11867243

[B14] Jensen HI Ammentorp J Kofoed PE User satisfaction is influenced by the interval between a health care service and the assessment of the service Soc Sci Med 2010 70 1882 1887 10.1016/j.socscimed.2010.02.035 20382459

[B15] Jensen HI Ammentorp J Kofoed PE Assessment of health care by children and adolescents depends on when they respond to the questionnaire Int J Qual Health Care 2010 22 259 265 10.1093/intqhc/mzq021 20427521

[B16] Kinnersley P Stott N Peters T Harvey I Hackett P A comparison of methods for measuring patient satisfaction with consultations in primary care Fam Pract 1996 13 41 51 10.1093/fampra/13.1.41 8671103

[B17] Bjertnaes OA Sjetne IS Iversen HH Overall patient satisfaction with hospitals: effects of patient-reported experiences and fulfilment of expectations BMJ Qual Saf 2011 in press 10.1136/bmjqs-2011-000137 21873465

[B18] Pettersen KI Veenstra M Guldvog B Kolstad A The Patient Experiences questionnaire: development, validity and reliability Int J Qual Health Care 2004 16 453 463 10.1093/intqhc/mzh074 15557355

[B19] Garratt AM Helgeland J Gulbrandsen P Five-point scales outperform 10-point scales in a randomized comparison of item scaling for the Patient Experiences Questionnaire J Clin Epidemiol 2011 64 200 207 10.1016/j.jclinepi.2010.02.016 20566267

[B20] Oltedal S Helgeland J Garratt A Pasienters erfaringer med døgnenheter ved soamtiske sykehus: metodedokumentasjon for nasjonal undersøkelse i 2006 ("Inpatient experiences with hospitals: documentation of methods for a national survey in 2006"), report 2-2007 Norwegian Knowledge Centre for the Health Services, Oslo

[B21] Elliott MN Edwards C Angeles J Hambarsoomians K Hays RD Patterns of unit and item nonresponse in the CAHPS Hospital Survey Health Serv Res 2005 40 2096 2119 10.1111/j.1475-6773.2005.00476.x 16316440 PMC1361246

[B22] Groves RM Fowler FJ Couper MP Lepkowski JM Singer E Tourangeau R Survey methodology 2009 2 Hoboken, New Jersey: Wiley

